# The Secretome of Preconditioned Mesenchymal Stem Cells Drives Polarization and Reprogramming of M2a Macrophages toward an IL-10-Producing Phenotype

**DOI:** 10.3390/ijms23084104

**Published:** 2022-04-07

**Authors:** Michelle Holthaus, Nivethiha Santhakumar, Thorsten Wahlers, Adnana Paunel-Görgülü

**Affiliations:** Department of Cardiothoracic Surgery, Heart Center, University of Cologne, 50937 Cologne, Germany; michelle.holthaus@uk-koeln.de (M.H.); nivethiha.santhakumar@gmail.com (N.S.); thorsten.wahlers@uk-koeln.de (T.W.)

**Keywords:** mesenchymal stem cells, secretome, macrophage polarization, preconditioning, immunosuppression

## Abstract

The preconditioning of mesenchymal stem cells (MSCs) has been recognized as an attractive tool to improve their regenerative and immunomodulatory capacities based on their paracrine effects. In this study, we examined the potential of an MSC-conditioned medium (MSC-CM) to alter the phenotype of murine macrophages and to drive reprogramming toward an anti-inflammatory, M2-like state in vitro. We further explored the impact of MSC cytokine preconditioning on the immunosuppressive properties of the MSC secretome. The MSC-CM suppressed the expression of proinflammatory genes in murine M1 macrophages, but only the CM from preconditioned MSCs (preMSC-CM) downregulated their expression during M1 polarization. Remarkably, only the preMSC-CM significantly increased the expression of M2a-, M2b- and M2c-specific genes and proteins during M2a polarization. Further, macrophages were found to secrete high levels of anti-inflammatory IL-10. Similarly, M2a macrophages cultured in the presence of the preMSC-CM displayed an enhanced expression of M2b/M2c-specific markers, suggesting that the secretome of preMSC promotes the repolarization of M2a-like macrophages to M2b/M2c subtypes. The preMSC-CM was found to be enriched in molecules involved in M2 polarization. Additionally, a unique downregulation of extracellular matrix components was observed. Altogether, the preMSC-CM may provide an attractive strategy to dampen inflammation by suppressing the expression of proinflammatory mediators and promoting the polarization and phenotype switch of M2a cells to IL-10-secreting M2b/M2c-like macrophages.

## 1. Introduction

Therapies using mesenchymal stem cells (MSCs) offer a promising treatment option in the field of regenerative medicine [1]. MSCs represent a heterogeneous group of cells mainly obtained from bone marrow, adipose tissue and umbilical cord blood playing a fundamental role in tissue repair and regeneration. In addition, they have gained a significant interest due to their immunomodulatory capacity [2]. Due to their low immunogenicity, these cells can be used in allogenic settings. However, the therapeutic potential of MSC transplantation is limited by the low ability of transplanted cells to engraft, home, survive and differentiate [3], as well as by the recruitment and migration of systemically administered MSCs to tumors [4]. As it has become evident that the therapeutic benefit of exogenously delivered MSCs is mainly due to their secretory properties [5], attention has now shifted to the MSC-derived secretome, which is now considered as a potential replacement for MSCs in cell therapy [6]. Proteomic analyses of an MSC-conditioned medium (MSC-CM) revealed that the cell-free secretome of MSCs is enriched with a large number of biologically active molecules, including cytokines, chemokines, growth factors, immunomodulatory molecules, exosomes and microvesicles [7,8]. The regenerative and immunomodulatory effects of the MSC secretome have led to its use in the treatment of a variety of diseases including myocardial infarction [9,10], liver [11] and lung [12] injury. It is well established that the anti-inflammatory effects of the MSC-CM are largely mediated by soluble immunoregulatory and anti-inflammatory molecules, such as IL-6, tumor growth factor (TGF)-β and prostaglandin E2 (PGE2) [13]. The paracrine action of MSCs is known to be influenced by the microenvironment and cells need to be licensed at the site of inflammation to activate the immunomodulatory activity [14]. In light of this, in vitro preconditioning of MSCs by strategies including 3D culture, hypoxia and inflammatory cytokines, significantly improved their immunomodulatory and regenerative properties [15].

Macrophages are characterized by their plasticity and diversity and play an important role in the initiation, development and resolution of the inflammatory response. They can be polarized into classically activated M1 macrophages and alternatively activated M2 macrophages, depending on the stimuli and signals from the microenvironment [16]. M2 macrophages are more heterogeneous and are subdivided into M2a, M2b and M2c subtypes. Notably, M2b macrophages regulate and dampen inflammatory responses and possess protective roles under noninfectious diseases, e.g., cardiovascular diseases. Thus, the modulation of macrophage polarization and activation of M2b cells has been proposed as a promising therapeutic tool in inflammatory diseases [17].

In the current study, we investigated the immunomodulatory efficacy of the secretome from bone marrow-derived MSCs with or without cytokine-preconditioning in terms of its potential to promote macrophage polarization and repolarization toward an anti-inflammatory phenotype. In addition, we performed proteomics to identify potential immunomodulatory molecules secreted by MSCs.

## 2. Results

### 2.1. The Secretome of Preconditioned MSCs Influences the Polarization of Macrophages under M1- and M2a-Polarizing Culture Conditions

Murine bone marrow-derived macrophages (M0) were polarized to an M1-like and M2a-like phenotype as described in the Materials and Methods section. M1 macrophages expressed significantly elevated levels of CD86 and reduced levels of CD206 when compared to M2a-like cells. Accordingly, M1 macrophages displayed increased expression of the proinflammatory genes *iNOS*, *IL-6* and *TNF-α* and reduced expression of M2-specific *Arg I* (Supplementary Figure S1).

MSCs isolated from the bone marrow of C57/BL6J mice were previously characterized in detail by our group [18]. The preconditioning of MSCs by IFN-γ and IL-1β [19] significantly increased the secretion of nitric oxide (NO) after 24 h of stimulation. Importantly, the further culturing of cells in the absence of cytokines for an additional 24 h did not result in a reduction of extracellular NO levels, accompanied by increased *iNOS* expression (Supplementary Figure S2).

**Figure 1:**
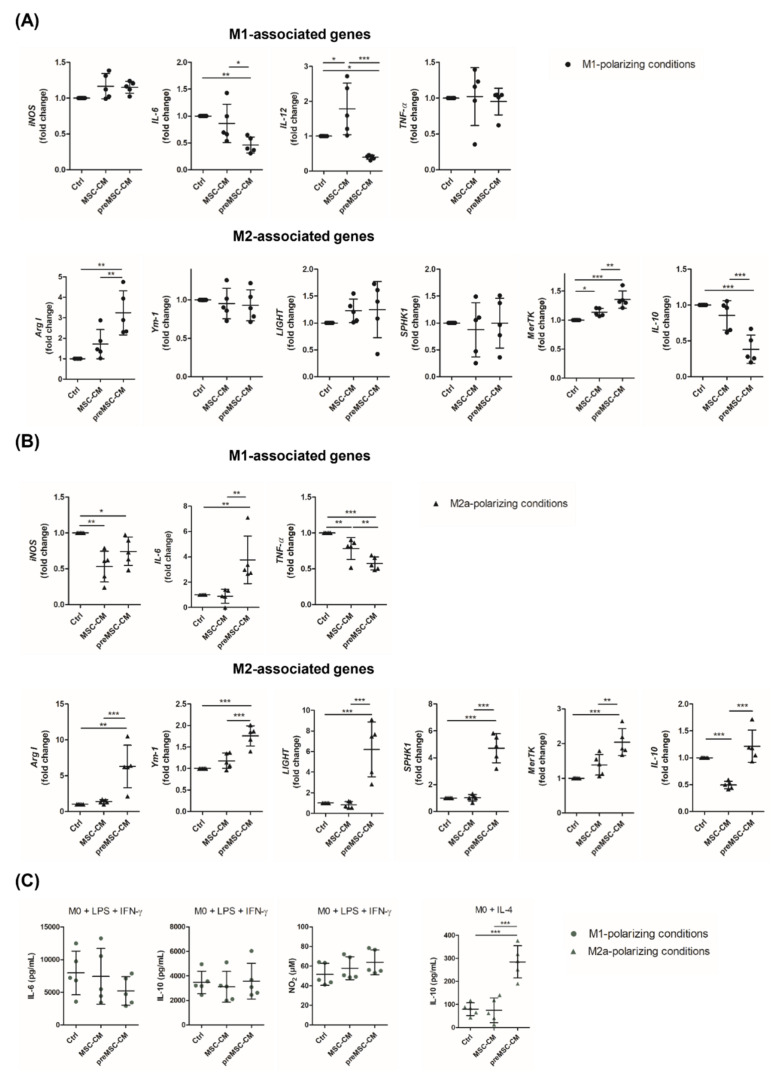
Effect of MSC-CM and preMSC-CM on macrophage polarization. Bone marrow-derived macrophages were cultured under M1-polarizing or M2a-polarizing culture conditions in medium supplemented with (pre)MSC-CM (20%) for 24 h. Cells cultured in the presence of cytokines but without (pre)MSC-CM served as control (Ctrl). The expression of M1- and M2-specific genes during M1 polarization (**A**) as well as during M2a polarization (**B**) was evaluated by real-time PCR (*n* = 5). (**C**) Cytokine quantification in culture supernatants was performed by Elisa. Nitrite levels were quantified by Griess assay (*n* = 5). * *p* < 0.05; ** *p* < 0.01; *** *p* < 0.001.

To investigate if the MSC-derived secretome was able to alter macrophage polarization, differentiated macrophages (M0) were treated with M1- and M2a-polarizing cytokines in the presence of 20% culture supernatant of unstimulated (MSC-CM) or preconditioned MSCs (preMSC-CM), respectively. As shown in Figure 1A, a significant downregulation of *IL-6*, *IL-12*, *IL-10* expression and upregulation of *Arg I* and *MerTK* was found in cells cultured in a medium supplemented with the preMSC-CM and M1-polarizing cytokines. Further, under M2a-polarizing conditions, the preMSC-CM induced a profound upregulation of the M2 marker *Arg I*, the M2a marker *Ym-1*, the M2b-specific genes *SPHK1* and *LIGHT* [20] as well as *MerTK*, which is highly expressed by M2c cells [21], compared to gene expression levels in cells treated with cytokines only and those cultured in the presence of the MSC-CM (Figure 1B). Notably, the expression of the proinflammatory genes *iNOS* and *TNF-α* was found to be reduced in the presence of the (pre)MSC-CM supporting the suppression of a proinflammatory macrophage phenotype. At the same time, upregulation of *IL-6* and *IL-10* was detected in these cells. However, the transcriptomic alterations in macrophages cultured under M1-polarizing conditions were not accompanied by changes in IL-6, IL-10 or NO_2_ levels (Figure 1C). In contrast, a marked secretion of IL-10 by macrophages stimulated with IL-4 and the preMSC-CM was detected indicating a shift toward an anti-inflammatory M2b/M2c phenotype [20] (Figure 1C).

### 2.2. The Secretome of Preconditioned MSCs Diminishes the Expression of Proinflammatory Cytokines in M1 Cells and Promotes Reprogramming of M2a-like Cells toward an IL-10-Producing Phenotype

**Figure 2:**
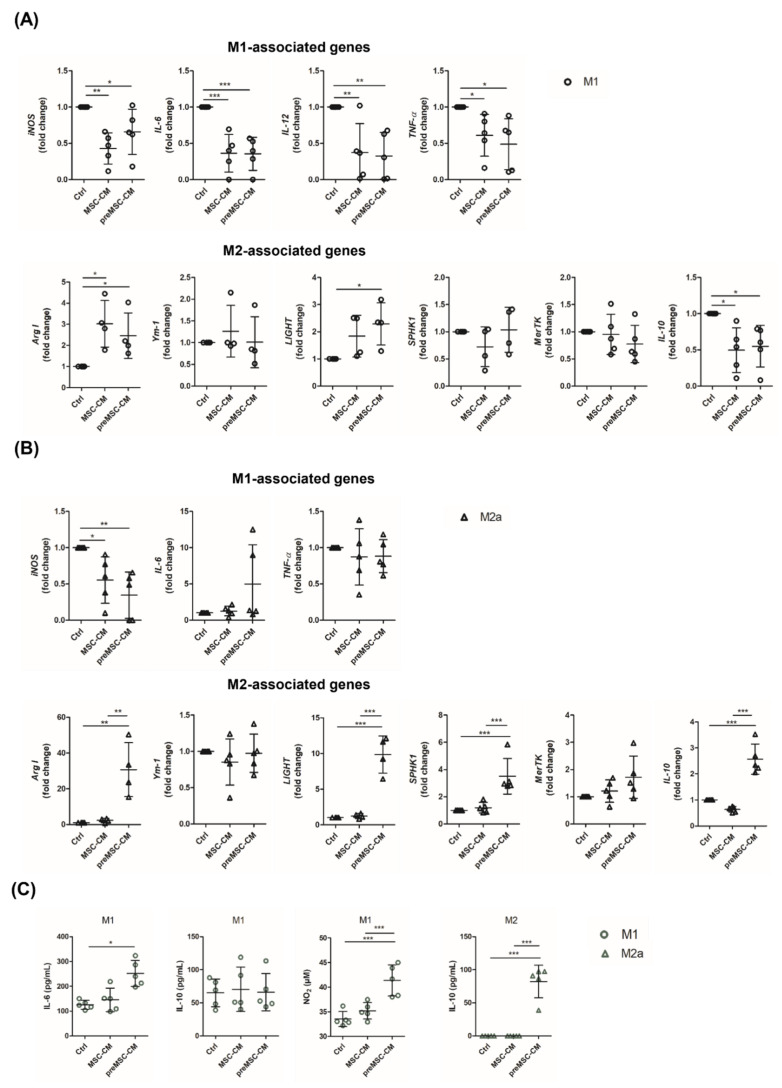
Effect of MSC-CM and preMSC-CM on macrophage reprogramming. Bone marrow-derived macrophages were polarized toward an M1-like or M2a-like phenotype for 24 h. Then, the medium was replaced and 20% (pre)MSC-CM was added to the culture medium. Cells were incubated for an additional 24 h. Macrophages cultured in the absence of (pre)MSC-CM served as control (Ctrl). The expression of M1- and M2-specific genes was analyzed in M1-polarized (**A**) and M2a-polarized (**B**) macrophages at the end of the incubation time (*n* = 4–5). (**C**) IL-6 and IL-10 were quantified in culture supernatants by Elisa. Nitrite levels were determined by Griess assay (*n* = 5). * *p* < 0.05; ** *p* < 0.01; *** *p* < 0.001.

Next, we questioned if soluble factors secreted by MSCs can alter the phenotype of polarized M1- and M2a-like macrophages. For this, cells were incubated in the presence of 20% (pre)MSC-CM for 24 h. Interestingly, both the secretome of MSCs and preMSCs reduced the expression of *iNOS*, *IL-6*, *IL-12*, *TNF-α*, *IL-10* and increased the expression of the M2-specific gene *Arg I* in M1 macrophages (Figure 2A). On the other hand, only the preMSC-CM enhanced the expression of *Arg I*, *LIGHT*, *SPHK1*, *IL-10* in M2a-polarized cells, resulting in significantly elevated IL-10 levels in the culture supernatants (Figure 2B,C). The proinflammatory gene *iNOS* was significantly downregulated whereas a visible, statistically not significant increase in *IL-6* expression was observed under these culture conditions. Furthermore, despite diminished *IL-6* and *iNOS* expression in M1 macrophages treated with the preMSC-CM, increased levels of IL-6 and nitrite were detected (Figure 2C).

**Figure 3:**
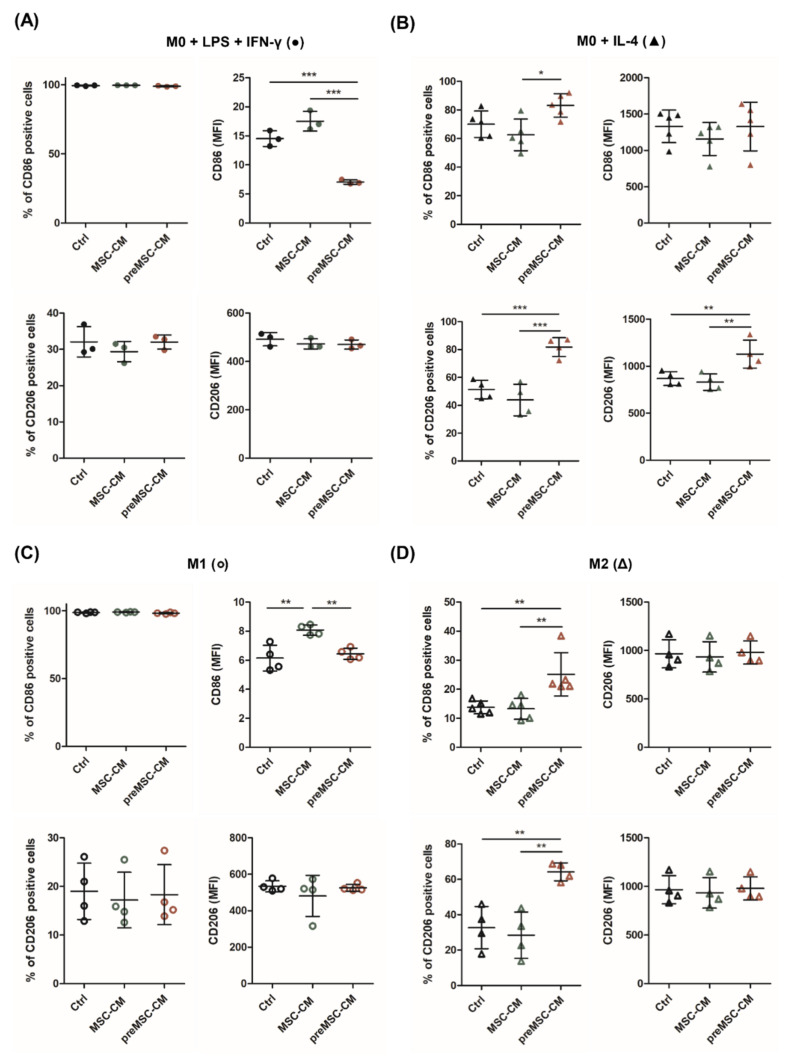
Effect of MSC-CM and preMSC-CM on the macrophage markers CD86 and CD206. Surface expression of CD86 and CD206 on macrophages cultured under (**A**) M1- and (**B**) M2a-polarizing culture conditions and 20% (pre)MSC-CMs was determined by flow cytometry. In addition, protein expression on polarized M1 (**C**) and M2 (**D**) macrophages was determined. Cells cultured in the absence of (pre)MSC-CM served as control (Ctrl). (*n* = 3–5). * *p* < 0.05; ** *p* < 0.01; *** *p* < 0.001.

These results imply that preMSC-secreted factors impede M1 polarization. Moreover, preMSC-CM promotes M2b/M2c polarization in the presence of IL-4 and additionally drives phenotype change in M2a macrophages. To prove these assumptions, we analyzed the expression of M1- and M2-specific markers more deeply. The secretome of preMSCs, but not of unstimulated MSCs, significantly decreased the expression of the M1 marker CD86 on macrophages treated with M1-polarizing cytokines, but did not change expression levels of the M2 marker CD206 (Figure 3A). In turn, preMSC-CM increased the number of CD86^+^ and CD206^+^ cells under M2a-polarizing conditions, confirming polarization toward an M2b/M2c-like phenotype (Figure 3B).

The treatment of M1 macrophages with the preMSC-CM also resulted in the downregulation of CD86 surface expression but did not change CD206 expression (Figure 3C). In contrast, the number of CD86^+^ and CD206^+^ cells strongly increased in M2a-polarized macrophages cultured in the medium supplemented with the preMSC-CM (Figure 3D). Representative dot plots are displayed in Supplementary Figure S3.

Overall, these results demonstrate that the preMSC-CM induces the polarization and reprogramming of M2 macrophages to an IL-10 secreting M2b/M2c-like phenotype.

### 2.3. Different Protein Abundance in the Secretome of preMSCs

**Figure 4:**
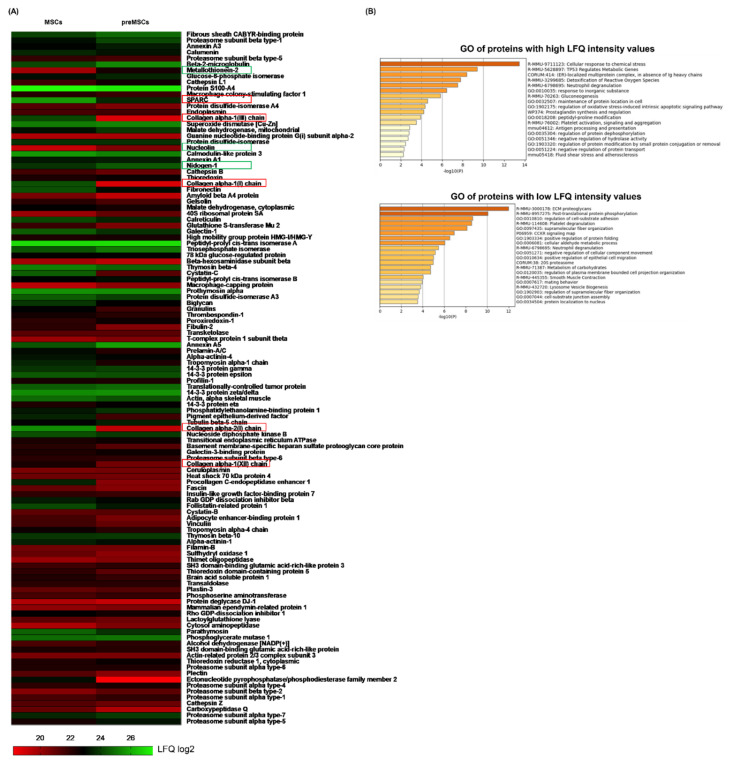
Characterization of secretome from MSCs and preMSCs. (**A**) Heat map of 116 peptides which were detected in the supernatants of MSCs and preMSCs on the basis of log2-transformed LFQ intensity values. (**B**) Gene ontology (GO) enrichment analysis of proteins found to be more or less enriched in the supernatants of preMSCs.

As we found that the property of MSCs to induce an anti-inflammatory M2b/M2c-like macrophage phenotype is strictly dependent on cytokine preconditioning, we next aimed to identify MSC-derived molecules, whose expression was specifically modified by cytokine preconditioning. To this end, a proteomic analysis was performed. MSC supernatants were first concentrated using centrifugal filters with a 3 kDa cutoff. Of note, the sample concentration did not result in an increase of protein amount suggesting that MSC-derived molecules are widely packaged into microvesicles and exosomes, respectively. Indeed, the sonication of the MSC-CM prior to centrifugation resulted in a ~sevenfold increase in protein concentration (data not shown). The exclusion of law molecular weight peptides did not affect the biological activity of the secretome as demonstrated by upregulated *LIGHT*, *SPHK1*, *MerTK* and *IL-10* expression in macrophages incubated with IL-4 and concentrated the preMSC-CM (Supplementary Figure S4). A subsequent LC–MS/MS analysis identified 116 peptides in the secretome of MSCs that were not detected in the medium control. Based on log2-transformed label-free quantitation (LFQ) intensity values, 51 peptides were found to be enriched in the secretome of preMSCs while 65 peptides were less abundant. MSC-specific protein secretion was visualized on a heat map (Figure 4A). All proteins detected were common for MSCs with and without preconditioning. Our results indicate that preMSCs express and secrete higher levels of metallothionein-2 (*Mt2*), nucleolin (*Ncl*) and nidogen-1 (*Nid1*). Interestingly, extracellular matrix (ECM) components, such as collagen alpha-1(III) chain (*Col3a1*), collagen alpha-1(I) chain (*Col1a1*), collagen alpha-2(I) chain (*Col1a2*) and collagen alpha-1(XII) chain (*Col12a1*) were markedly reduced in the secretome of preMSCs. The gene ontology (GO) enrichment of proteins upregulated by preconditioning revealed functional clusters belonging to cellular response to chemical stress, metabolic pathways, oxidative stress, neutrophil degranulation and the regulation of protein modification and activation. Downregulated proteins were mainly enriched in biological processes such as extracellular matrix organization, protein folding degradation and phosphorylation as well as cell migration (Figure 4B). Thus, the secretome of preMSCs substantially differs from that of MSCs.

## 3. Discussion

Due to their extraordinary plasticity, macrophages have acquired a lot of interest as potential therapeutic targets in inflammatory disorders. Recent studies have demonstrated that MSC-derived mediators promote the polarization of human and murine macrophages toward an anti-inflammatory, reparative M2 phenotype [22,23]. In the present study, we studied the immunosuppressive potency of the secretome derived from MSCs with or without cytokine preconditioning on murine macrophages in vitro.

We confirmed that the MSC secretome markedly suppresses the expression of CD86 and proinflammatory factors in M1-polarized murine macrophages, although no upregulation of anti-inflammatory IL-10 was observed [24], but rather a significant downregulation of IL-10 gene expression. As IL-10 expression becomes regulated by LPS [20], this downregulation may indicate a secretome-mediated amelioration of LPS signaling pathways. Although *Arg I* expression increased markedly in the presence of the (pre)MSC-CM, no reduction in extracellular nitrite levels was found, showing that the expression of arginase I does not result in an immediate restriction of L-arginine availability, which represents a substrate for iNOS. Unexpectedly, in M1 macrophages cultured in the presence of the preMSC-CM, we found an increase in nitrite levels despite a suppressed *iNOS* expression. However, it is possible, that the downregulation of iNOS activity is delayed. Moreover, IL-6 secretion was enhanced by the preMSC-CM. In this regard, increased IL-6 levels do not mandatorily promote inflammation, but rather may enhance polarization toward an M2-like phenotype [25]. Thus, the MSC secretome efficiently attenuates the expression of proinflammatory genes in M1 macrophages, independently of MSC preconditioning. The secretome of preMSC significantly reduced CD86 expression on M1 cells, which was found to be enhanced by the MSC-CM. Thus, in contrast to previously published reports [26,27], the (pre)MSC secretome does not seem to support a phenotypic switch to M2-like cells, as demonstrated by the widely unaltered expression of M2-related genes (*Ym-1*, *SPHK1*, *MerTK*) and CD206 protein.

Regarding the polarization process, only the preMSC-CM, but not the MSC-CM, inhibited M1 polarization, as shown by a reduced expression of CD86 and proinflammatory genes. Similar to the findings mentioned above, IL-10 secretion did not increase despite an upregulated *Arg I* and *MerTK* expression. MerTK plays a crucial role in the suppression of immune responses and is strongly upregulated on M2c macrophages, increasing their ability to clear apoptotic bodies [21]. Against this background, it is likely that an increased *MerTK* expression in macrophages may facilitate the removal of apoptotic cells, although this hypothesis has not yet been tested. Our results have further shown that the preconditioning of MSCs with proinflammatory cytokines represents a prerequisite for their capability to drive polarization toward an anti-inflammatory, regulatory macrophage phenotype. In fact, the preMSC-CM strongly upregulated the expression of the M2a-specific gene *Ym-1* as well as of the M2b- and M2c-specific genes *LIGHT*, *SPHK1*, *IL-6*, *MerTK*, *IL-10*, and simultaneously enhanced the protein expression of IL-10, CD86 and CD206 during M2a polarization. Whereas CD86 is considered as a marker of the M2b subtype, CD206 is rather specific for M2a and M2c macrophages [17]. The upregulation of CD206 and *MerTK* further suggests that preMSC-induced macrophages may display an overall increased phagocytic capacity, supporting their contribution to the resolution of inflammation [28]. However, as the ability of macrophages to produce high levels of IL-10 is attributed to a regulatory phenotype [29,30], we conclude that the preMSC-CM induces polarization toward an anti-inflammatory macrophage phenotype sharing characteristics with the M2b/M2c subtypes. Similarly, M2a macrophages were found to undergo reprogramming to an M2b/M2c-like phenotype after treatment with the preMSC-CM. These cells displayed increased mRNA levels of *Arg I*, *LIGHT*, *SPHK1*, *IL-10* as well as upregulation of IL-10, CD86 and CD206 expression.

Altogether, our results are largely in line with previously reported data, including those from our own study, demonstrating an increased IL-10 secretion by macrophages after treatment with (pre)MSCs [18,31,32]. They additionally demonstrate that the immunoregulatory effects solely depend on the preMSC-derived secretome.

At present, several MSC-derived factors involved in the suppression of immune responses have already been identified [33]. In this regard, a previous work from our group revealed that the M2b polarization of macrophages in the presence of preMSCs strongly depends on IL-6 signaling, although we could not exclude the involvement of additional mediators [18]. Here, we performed mass spectrometry and identified various factors previously described to promote macrophage polarization. Among them, metallothionein-2 [34], cathepsin [35], nucleolin [36], nidogen-1 [37], thioredoxin [38], and annexin A5 [39] were demonstrated to be involved in M2/M2b polarization. Of note, these factors were found to be of higher abundance in the secretome of preMSCs when compared with the secretome of MSCs, indicating that they might indeed be implicated in the reprogramming of M2a macrophages toward anti-inflammatory M2b/M2c-like cells. In addition, SPARC, which has already been reported to improve M1 polarization [40], was markedly reduced in the secretome of preMSCs.

Another interesting finding of our study was that preconditioning strongly suppressed profibrotic pathways in MSCs, accompanied by a strong downregulation of ECM components (collagen alpha-1(I)chain, collagen alpha-2(I) chain, collagen alpha-1(XII) chain, procollagen C-endopeptidase enhancer 1, fibronectin, biglycan, fibulin-2). This suggests that the enhanced immunosuppressive features of preMSCs correlate with a less fibrotic phenotype making these cells probably less attractive for regenerative strategies. However, it is actually not clear if the downregulation of ECM proteins contributes to M2b/M2c polarization. In this respect, Sapudom et al. proposed that collagen fibril intensity increases the expression of some proinflammatory cytokines in polarized THP1 macrophages and thus their proinflammatory characteristics [41].

Our study has several limitations: First, it is widely accepted that primary MSCs may display functional heterogeneity depending on donors’ age, their anatomic origin as well as in vitro expansion. Consequently, their secretome profile may vary significantly [42]. The use of standardized MSCs derived from induced pluripotent stem cells may represent a feasible strategy to omit these limitations and to overcome their restriction for clinical use. Second, as this study was performed in vitro, additional studies using animal models are mandatory to validate these data and to prove the potential relevance for humans. Third, we did not consider the impact of MSC-derived exosomes or microvesicles on macrophage polarization. In this respect, numerous studies have already confirmed that MSC-derived exosomes promote the polarization of macrophages to the M2 phenotype [43,44,45]. Finally, in our proteomic analysis, we used a pool of MSC secretome instead of replicates, so that no statistical significance of the differences could be assessed. Therefore, these results should generally be interpreted with caution. However, microarray data previously published by our group [46] demonstrated a strong upregulation of *Mt2*, *Nid1* and downregulation of *SPARC*, *Col3a1*, *Col1a2*, *Col12a1*, *Pcolce* in preMSCs, thus strongly supporting the data presenting herein.

In conclusion, we demonstrate that the secretome of preconditioned bone-marrow-derived MSCs suppresses the expression of proinflammatory cytokines in M1 macrophages as well as under M1-polarizing culture conditions. PreMSC-derived soluble factors did not skew the M1 phenotype to an M2-like phenotype under these conditions. Otherwise, the preMSC secretome consists of a complex cocktail of factors that stimulate macrophage polarization and the reprogramming of M2a cells toward an IL-10-producing, M2b/M2c-like phenotype. In the light of our data showing a reduced content of ECM components in the secretome of preMSCs, we suggest that the secretome of these cells may be useful for the treatment of inflammatory disorders but not for regenerative therapies enclosing tissue engineering.

## 4. Materials and Methods

### 4.1. Animals

Adult 8–12-week-old female C57/BL6J mice bred on the Animal Facility of the University of Cologne (Germany) were used for all experiments. Mice were maintained in the local animal facility at a 12 h light/dark cycle with food and water ad libidum.

### 4.2. Isolation and Preconditioning of Bone-Marrow-Derived MSCs

Mice were euthanized by cervical dislocation. Bone marrow cells were obtained by flushing out femurs with PBS. Cells were cultured in a density of 1.5–2 × 10^6^/cm^2^ in an MSC culture medium (Pan-Biotech, Aidenbach, Germany) supplemented with 2.5 ng/mL human basic fibroblast growth factor FGF (FGF-b, Peprotech, Hamburg, Germany), 100 U/mL penicillin, 10 µg/mL streptomycin (Sigma Aldrich, St. Louis, MA, USA) at 37 °C until a proliferative and homogenous MSC population was obtained. MSCs were characterized phenotypically by flow cytometry and by their potential to differentiate into chondrocytes, adipocytes and osteoblasts [18]. Bone marrow-derived MSCs of passage 9 were used in this study.

For preconditioning, MSCs were kept in an MSC culture medium supplemented with 2.5 ng/mL FGF-b until cells reached a 70–80% confluency. MSCs were stimulated with 30 ng/mL recombinant murine IFN-γ (Peprotech, Hamburg, Germany) and 3 ng/mL recombinant murine IL-1β (Peprotech, Hamburg, Germany) for 24 h. MSCs without cytokine treatment served as control. Then, cells were washed twice with PBS to remove any residual factors and serum and further cultured in RPMI 1640 medium (Pan-Biotech, Aidenbach, Germany) supplemented with 100 U/mL penicillin and 10 µg/mL streptomycin (Merck, Darmstadt, Germany). Twenty-four hours later, culture supernatants were centrifuged to remove cellular debris. Culture supernatants from three different MSCs cultures (with or without preconditioning) were pooled, aliquoted and stored at −80 °C until further use. In some experiments, pooled culture supernatants were concentrated ~4-fold by a centrifugal filter device (3 kDa cutoff; Thermo Fisher, Waltham, MA, USA) and stored frozen at −80 °C. Pooled MSC-CM or concentrated MSC-CM, respectively, were used for the cell culture experiments.

### 4.3. Isolation and Polarization of Murine Macrophages

For the preparation of murine bone marrow-derived macrophages, mice were sacrificed and femur and tibias were flushed with PBS, followed by red blood cells lysis using RBC Lysis Buffer (Invitrogen, Waltham, MA, USA). Bone marrow cells were cultured in 6-well plates in an RPMI medium supplemented with 20% FCS containing 20 ng/mL recombinant murine M-CSF (Peprotech, Hamburg, Germany), 100 U/mL penicillin and 10 µg/mL streptomycin (Merck, Darmstadt, Germany) at a density of 0.25 × 10^6^ cells/mL for seven days. On day 3 and day 6, the medium was replaced by fresh medium. For M1 polarization, differentiated macrophages (M0) were cultured in an RPMI medium with 10% FCS supplemented with 20 ng/mL recombinant murine IFN-γ (Peprotech, Hamburg, Germany) and 100 ng/mL LPS (Merck, Darmstadt, Germany) for 24 h. To induce polarization into an M2a-like phenotype, macrophages were cultured in a medium supplemented with 20 ng/mL recombinant murine IL-4 (Peprotech, Hamburg, Germany).

### 4.4. Quantification of Nitric Oxide (NO) Levels

Nitric oxide (NO) production was quantified by measuring nitrite (NO_2_) in culture supernatants, using a modified Griess reagent (Sigma Aldrich). In brief, 80 µL of supernatant was mixed with 80 µL of Griess reagent and incubated at room temperature for 15 min. Absorbance at 540 nm was measured using a microplate reader (FLUOstar Omega, BMG Labtech, Ortenberg, Germany) and nitrite concentrations were estimated using a standard nitrite curve (range 0 µM–100 µM).

### 4.5. Real-Time PCR

Total RNA was extracted using an RNeasy Mini Kit (Qiagen, Hilden, Germany) according to the manufacturer’s instructions. RNA was reverse transcribed using a High Capacity cDNA Reverse Transcription Kit (Applied Biosystems, Waltham, MA, USA). Real-time PCR was performed using Power SYBR Green PCR Master Mix (Applied Biosystems) and specific primers for *IL-6*, *IL-10*, *IL-12* [47], *Arg I*, *iNOS*, *Ym-1*, *SPHK1*, *LIGHT*, *MerTK* [18] and *TNF-α* [48]. All samples were run in triplicates. The expression of target genes was normalized to the GAPDH housekeeping gene and expressed as fold change using the 2^−ΔΔCT^ method.

### 4.6. Flow Cytometry

Macrophages were incubated with TruStain FcX antibody (BioLegend, San Diego, CA, USA) for 10 min on ice to prevent unspecific binding of antibodies to Fc receptors. Then, cells were stained with rat anti-mouse CD86-APC (clone GL1, BioLegend, San Diego, CA, USA) or rat anti-mouse CD206-Brilliant Violet 421 (clone V068C2, BioLegend, San Diego, CA, USA) for 20 min at 4 °C in the dark and further washed with PBS supplemented with 2% FCS and 1 mM EDTA. Unstained cells were used as negative control for fluorescence. All data were collected by flow cytometry using FACSCanto II and DIVA software (Beckman Coulter, Brea, CA, USA).

### 4.7. ELISA

For quantification of IL-6 and IL-10, the Mouse IL-6 DuoSet ELISA and Mouse IL-10 DuoSet ELISA (R&D Systems, Minneapolis, MN, USA) were used. Cytokine quantification was performed by following the manufacturer’s instructions.

### 4.8. Proteomics

For the proteomic analysis, concentrated MSC supernatants were sonicated and centrifuged at 2500× *g* for 10 min at 4 °C. Supernatants were used to quantify protein concentration by a BCA assay (Thermo Fisher, Waltham, MA, USA). A total of 50 µg protein was solved in urea buffer (>6 M), reduced by 5 mM DTT and digested with Trypsin and LysC. Peptides were cleaned using C18 STAGE Tips. Liquid chromatography tandem mass spectrometric analysis (LC–MS/MS) of peptides was performed at the CECAD Proteomics Core Facility, Cologne, Germany.

All samples were analyzed on a Q Exactive Plus Orbitrap mass spectrometer coupled to an EASY nLC (both Thermo Scientific, Waltham, MA, USA). Peptides were loaded with solvent A (0.1% formic acid in water) onto an in-house packed analytical column (40 cm–75 µm I.D., filled with 2.7 µm Poroshell EC120 C18, Agilent, Santa Clara, CA, USA). Peptides were chromatographically separated at a constant flow rate of 250 nL/min using the following gradient: 4–6% solvent B (0.1% formic acid in 80% acetonitrile) within 5.0 min, 6–23% solvent B within 120.0 min, 23–54% solvent B within 7.0 min, 54–85% solvent B within 6.0 min, followed by washing and column equilibration. The mass spectrometer was operated in data-dependent acquisition mode.

The MS1 survey scan was acquired from 300–1750 *m*/*z* at a resolution of 70,000. The top 10 most abundant peptides were isolated within a 2.1 Th window and subjected to HCD fragmentation at a normalized collision energy of 27%. The AGC target was set to 5e5 charges, allowing a maximum injection time of 60 ms. Product ions were detected in the Orbitrap at a resolution of 17,500. Precursors were dynamically excluded for 20.0 s.

All mass spectrometric raw data were processed with Maxquant (v2.0.3.0) using default parameters. Briefly, MS2 spectra were searched against the murine canonical Uniprot FASTA (reference UP000000589, downloaded at 26 August 2020) database, including a list of common contaminants. False discovery rates on protein and PSM level were estimated by the target-decoy approach to 1% (protein FDR) and 1% (PSM FDR), respectively. The minimal peptide length was set to 7 amino acids and carbamidomethylation at cysteine residues was considered as a fixed modification. Further data handling was done in Perseus (v1.6.15.0). For the gene ontology (GO) term enrichment analysis, datasets were uploaded to Metascape (http://metascape.org, accessed on 19 January 2022) [49].

### 4.9. Statistical Analysis

Experimental data were analyzed with GraphPad Prism software (GraphPad Software, San Diego, CA, USA) and are reported as mean ± standard deviation (SD).

Statistical differences between groups were analyzed using a one-way ANOVA with Newman-Keuls post hoc test. A *p*-value of less than 0.05 was considered as statistically significant.

## Data Availability

The data presented in this study are available on request from the corresponding author.

## Supplementary Materials

The following supporting information can be downloaded at: https://www.mdpi.com/article/10.3390/ijms23084104/s1.
