# Supplementary material for: The Secretome of Preconditioned Mesenchymal Stem Cells Drives Polarization and Reprogramming of M2a Macrophages toward an IL-10-Producing Phenotype
Source: Int J Mol Sci. 2022 Apr 7;23(8):4104. doi: 10.3390/ijms23084104 (PMC9024470; doi:10.3390/ijms23084104)
Supplement: Supplementary file 1 [file ijms-23-04104-s001.zip › ijms-1594307-supplementary.pdf]

International Journal of
Molecular Sciences

MDPI

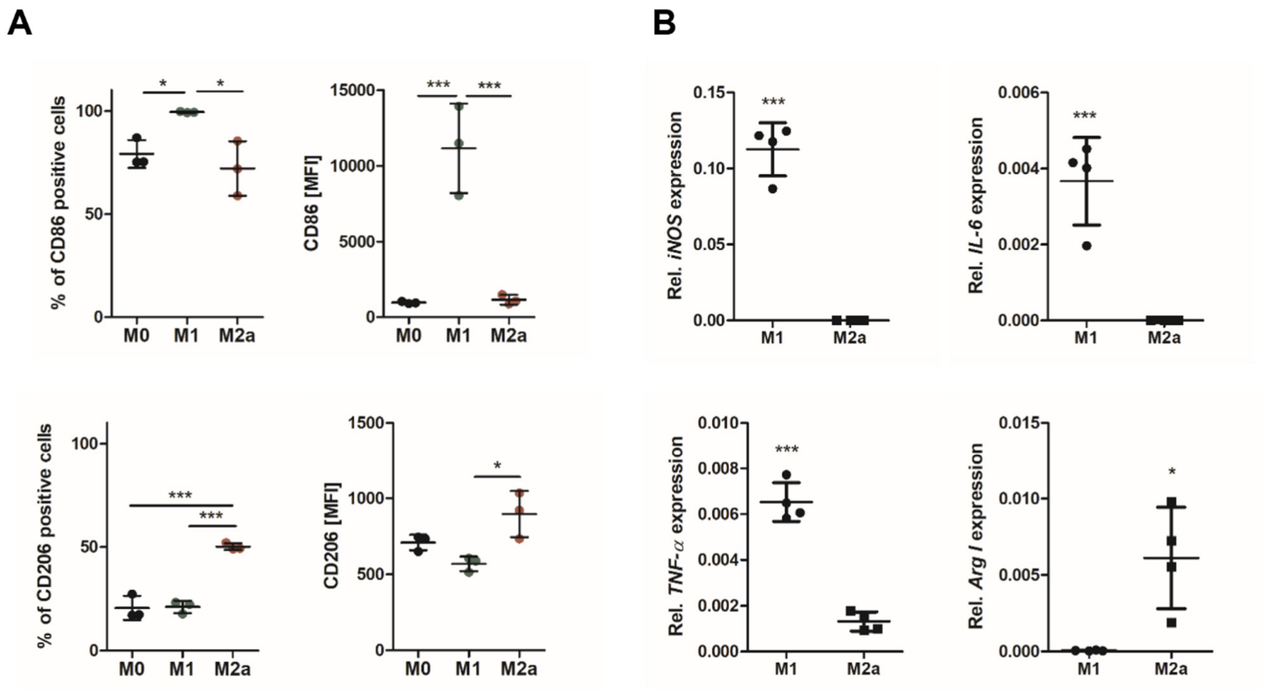

Figure S1. Characterization of polarized macrophages. Murine, bone marrow-derived cells were cultured in the presence of 20 ng/ml M-CSF for seven days to obtain differentiated M0 macrophages. Cells were further polarized into M1-like macrophages by LPS (100 ng/ml) and IFN-γ (20 ng/ml) or into M2-like cells by IL-4 (20 ng/ml). (**A**) After 24 h, the expression of CD86 (M1 marker) and CD206 (M2 marker) was quantified by flow cytometry. The percentage of positive cells and median fluorescence intensity (MFI) are depicted (n = 3). (**B**) The expression of the M1-specific genes *iNOS*, *IL-6* and *TNF-α* as well as of M2-specific *Arg I* were analyzed by real-time PCR (n = 4). *p < 0.05; ***p < 0.001.

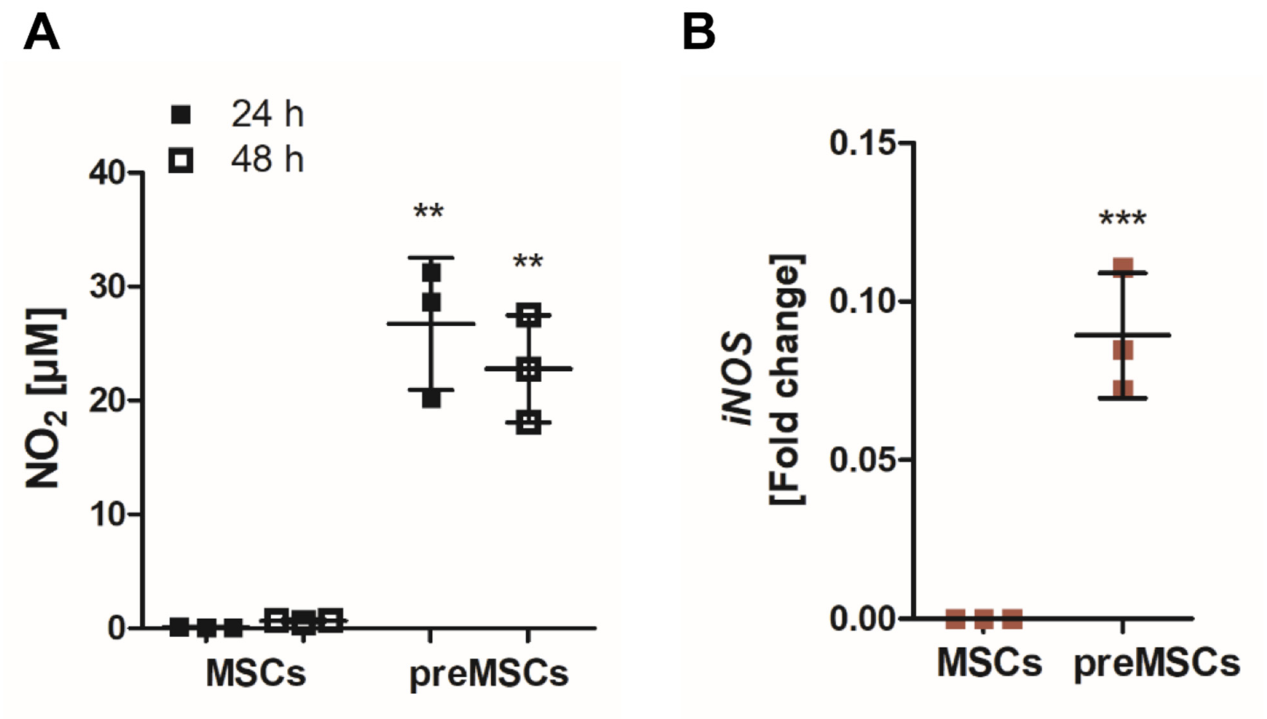

Figure S2. Impact of preconditioning on nitric oxide production by MSCs. Murine, bone marrow-derived MSCs were treated with IFN-γ (30 ng/ml) and IL-1β (3 ng/ml) for 24 h. Then, supernatants were collected. Cells were washed with PBS and further cultured in serum-free culture medium for additional 24 hours. (**A**) Nitrite levels were quantified in culture supernatants of MSCs and preconditioned MSCs (preMSCs) by Griess assay after 24 h and 48 h (n = 3). (**B**) The expression of *iNOS* was quantified by real-time PCR after 48 h (n = 3). **p < 0.01; ***p < 0.001.

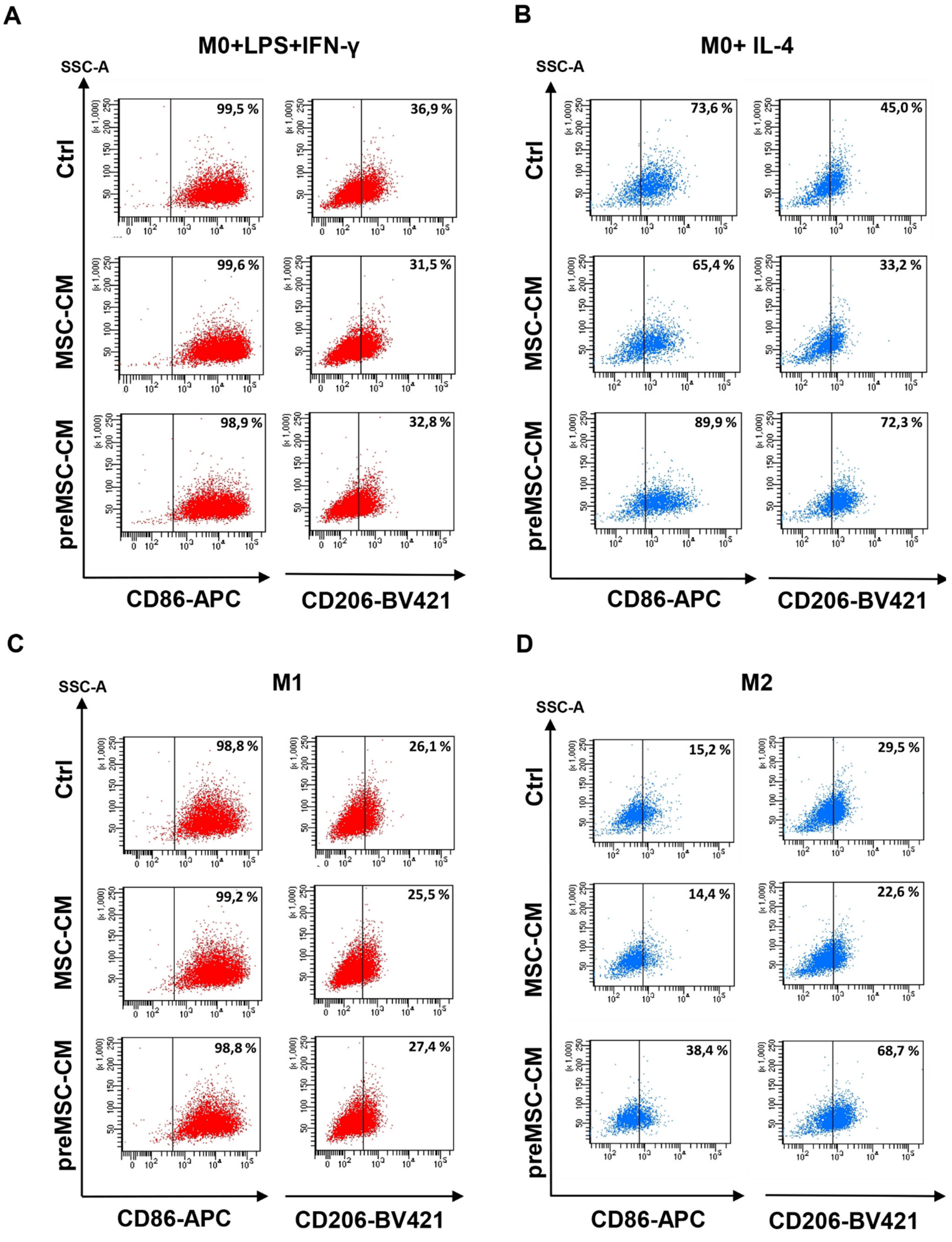

Figure S3. Impact of (pre)MSC-CM on CD86 and CD206 surface expression. Representative flow cytometry dot plots from one experiment showing CD86 and CD206 expression on macrophages

under M1- (**A**) and M2a-polarizing (**B**) culture conditions. In addition, CD86 and CD206 expression on M1 (**C**) and M2 (**D**) macrophages is depicted. Cells cultured in the absence of (pre)MSC-CM served as control (Ctrl).

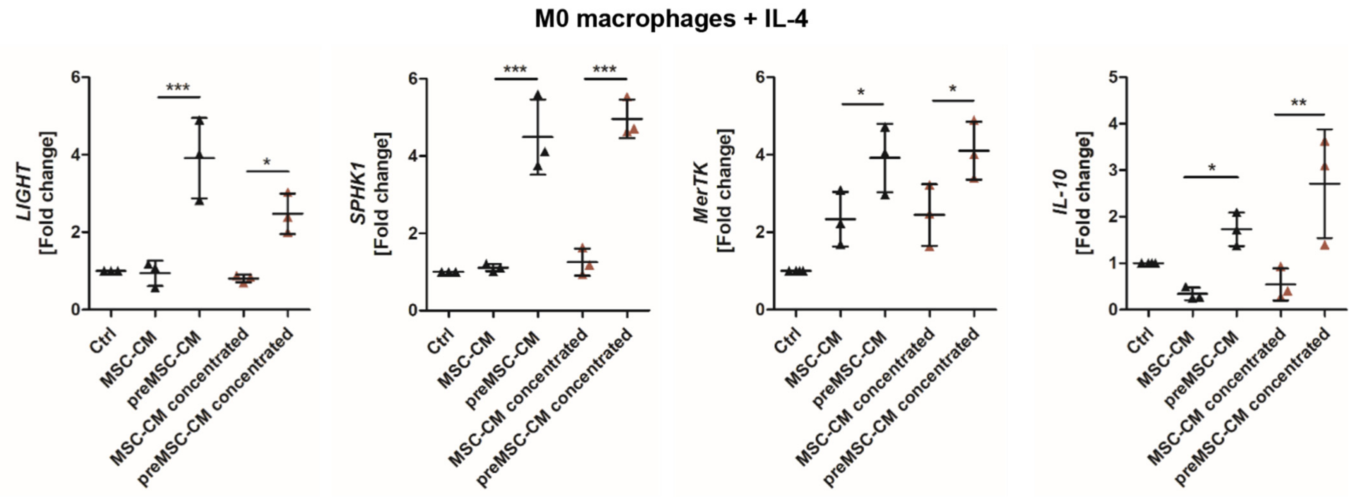

Figure S4. Impact of concentrated MSC-CM on the expression of M2b/M2c-specific genes in macrophages. Murine M0 macrophages were incubated in the presence of IL-4 (20 ng/ml) and 20% MSC-CM or preMSC-CM for 24 h. In parallel experiments, MSC-CM was 4-fold concentrated using centrifugal filters (3 kDa cut-off) and 5% concentrated CM was added to the cells. Cells cultured in the absence of MSC-CM served as control (Ctrl). The expression of the M2b/M2c-specific genes *LIGHT*, *SPHK1*, *MerTK* and *IL-10* was analyzed by real-time PCR (n = 3). *p < 0.05; **p < 0.01; ***p < 0.001.
